# Mediation effect of hope on the relationship between inner strength and self-management in patients after percutaneous coronary intervention

**DOI:** 10.3389/fpsyg.2024.1268598

**Published:** 2024-01-24

**Authors:** Shuhua Shang, Xuemei Zheng, Zhongmei Xu, Si Sun, Tianyi Huang

**Affiliations:** ^1^Department of Cardiology, Nanjing First Hospital, Nanjing Medical University, Nanjing, China; ^2^Department of Nursing, Nanjing First Hospital, Nanjing Medical University, Nanjing, China

**Keywords:** percutaneous coronary intervention, inner strength, hope, self-management, mediation analysis

## Abstract

**Background:**

Effective self-management can enhance a patient’s quality of life and delay disease progression. However, motivating patients to adhere to self-management behavior following percutaneous coronary intervention (PCI) remains a challenge. With the robust development of positive psychology and interdisciplinary research, the role of psychology factors in patients’ health behavior has increasingly garnered attention. This study, focusing on positive psychological qualities, aims to investigate the relationship between inner strength, hope, and self-management in patients post-PCI, and to analyze the mediating role of hope between inner strength and self-management.

**Methods:**

A cross-sectional survey was conducted among 216 PCI patients from a tertiary hospital in Nanjing. Research instruments included a self-designed general information questionnaire, the Inner Strength Scale (ISS), the Herth Hope Index (HHI), and the Coronary Self-Management Scale (CSMS). *T*-test, analysis of variance, Pearson’s correlation analysis, and mediating effect test were utilized for statistical analysis.

**Results:**

The average scores of the ISS, HHI, and CSMS were 81.46 ± 12.00, 35.94 ± 5.38, and 86.79 ± 14.84, respectively. Inner strength was positively correlated with hope and self-management (*r* = 0.867, *r* = 0.630, respectively; all *P* < 0.05), and hope was positively correlated with self-management (*r* = 0.671, *P* < 0.05). Moreover, hope had a complete mediating effect between inner strength and self-management (β = 0.630, *P* < 0.01).

**Conclusion:**

The inner strength, hope, and self-management of patients with PCI are at a moderate level. Inner strength primarily influences patients’ self-management behavior through hope, suggesting that medical staff can target hope to help patients build confidence in life after illness, form and accumulate inner strength, thereby promoting their self-management and improving prognosis.

## 1 Introduction

According to the Global Burden of Disease (GBD) Study 2019, approximately 523 million people worldwide suffer from cardiovascular disease, resulting in 18.6 million deaths. Coronary heart disease (CHD) accounts for about half of these deaths (Roth et al., 2020). CHD, a prototypical cardiovascular disease, poses a serious threat to human health, with China also facing a CHD crisis (Liu et al., 2019; Zhao et al., 2019). As reported in 2021, there are 11.39 million cases of coronary heart disease in China, and the incidence is expected to continue to rise due to multiple risk factors such as aging (Writing Committee of the Report on Cardiovascular Health and Diseases in China, 2022). With the significant progress of interventional techniques, percutaneous coronary intervention (PCI) has become a crucial strategy for revascularization in patients with CHD (Gao, 2019). According to the Heart Disease and Stroke Statistics from the American Heart Association, inpatient PCI procedures ranged from 1,310,000 to 480,000 annually between 2006 and 2016 (Lloyd-Jones et al., 2010; Tsao et al., 2023). In Germany, about 4,000,000 PCI procedures per year between 2014 and 2017 (Huber et al., 2020). There were 751,728 PCI procedures in persons aged 30 years and above between 2000/01 and 2020/21 in Australia (Kumsa et al., 2023). The database of the National Health Commission indicates that as of 2022, the total number of PCIs in China reached 1,293,932, with a mortality rate of 0.37% (Pan, 2023). PCI can effectively alleviate ischemic symptoms and reduce patient mortality (Hoole and Bambrough, 2020). However, while PCI can save the lives of patients with CHD, it struggles to reverse the pathological state of vessels. Patients remain in a “survival with disease” status post-PCI, and the risk of cardiovascular events remains high (Freites et al., 2022). Furthermore, existing systematic review indicates that there is no significant improvement in the quality of life for patients after PCI (Hirao et al., 2023).

Relevant guidelines suggest that self-care is a specific measure for secondary prevention of CHD, beneficial in reducing the occurrence of cardiovascular endpoint events (Knuuti et al., 2020; Liu et al., 2023). The World Health Organization (WHO) emphasizes that fostering positive self-management behaviors represents the most effective way to enhance the quality of life for individuals with chronic illnesses (World Health Organization [WHO], 2002). However, it is challenging for individuals to adjust their lifestyle and adhere to self-management behaviors (Aggarwal et al., 2021). Therefore, improving self-management compliance has become a research hotspot in the field of coronary heart disease.

Orem believed that self-care is a series of self-regulating behaviors individuals undertake to maintain their growth, development, and the integrity and normal function of their own structure (Orem, 1981). Self-management after PCI consists a range of behaviors, including smoking cessation, adopting a balanced diet, engaging in physical exercise, managing sleep, psychological adjustment, adherence to prescribed medications, self-monitoring of recurrent myocardial infarction and timely intervention (Peterson et al., 2014). Previous studies have shown that higher levels of self-management are associated with higher levels of quality of life and health promotion behaviors, and lower levels of readmission (Cheng and Kotronoulas, 2020; Collins et al., 2021). This suggests that improving self-management ability is not only key to promoting a healthy lifestyle, but also an important strategy to save medical costs and optimize the allocation of medical resources. However, existing studies have shown that the self-management status of patients post-PCI is not optimistic, and motivating patients to adhere to self-management behavior remains a difficult problem (Sua et al., 2020; Zhu et al., 2022). Riegel et al. (2012) proposed a middle-range theory of self-care for chronic illness, defining self-care in the context of chronic illness as the process of maintaining health through the practice of health promotion and disease management. This theory identifies two underlying mechanisms in self-care for chronic illness: reflection and decision-making. Reflection helps patients understand and master the knowledge, skills, and principles of chronic illness management, while decision-making involves actions taken by patients after assessing the context of disease management and the coping resources available to them. Thus, effective self-care not only requires a grasp of health management knowledge but also demands sufficient behavioral motivation. Empirical findings draw similar conclusions that health education is a key component of behavior change, but general health education alone has been shown to be insufficient to motivate self-management in patients with CHD (Feldman and Sills, 2013; Peterson et al., 2014).

With the rise of positive psychology and the deepening of the “bio-psychological-social” modern medical model, the influence of internal potential on health behavior is gaining attention (Müller et al., 2022). Promoting patient behavior change through intrinsic potential and advantages may be a new approach to optimizing patient self-management after PCI.

Inner strength (IS), defined as a universal reaction where patients grow and gain strength from adversity, is considered a core resource for humans to overcome disease and promote health (Boman et al., 2015). In the early 2000s, Roux conducted a meta-integration of relevant qualitative studies and formed the middle-range Theory of Inner Strength (TIS) (Roux et al., 2002). The interpretive framework comprises four processes: (1) experiencing the pain brought by illness and finding meaning in life experiences (2) establishing connections with oneself, family, friends, and spiritual power (3) actively participating in the decision-making process; (4) taking proactive actions. Through the process of experiencing illness, individuals can gradually develop an inner strength. The TIS posits that by enhancing inner strength, individuals can better utilize internal and external resources, make informed choices and decisions, and effectively cope with challenging events, thereby improving self-management. Unlike the risk- and disease-based medical service model, the TIS focuses on tapping into a patient’s potential during illness, mobilizing intrinsic motivation for health promotion, and improving self-management (Viglund et al., 2013). However, most inner strength studies in the medical field focus on the elderly and tumor patients, with few focusing on CHD patients (Viglund et al., 2014; Boman et al., 2015; Smith et al., 2019). In terms of research methods, more qualitative studies are used to understand the experience of inner strength. Only Dingley showed that inner strength is an important predictor of self-management in female cancer patients through a cross-sectional study (Dingley and Roux, 2014). There is a lack of quantitative studies on inner strength in post-PCI patients, and limited information is available on the mechanisms through which the inner strength affects self-management (Dingley et al., 2001; Mendes et al., 2010).

Previous evidence has suggested that inner strength is a significant predictor of hope (Gottlieb, 2014). Patients find meaning in life, make new connections, and take positive action during their illness, thus feeling hopeful about the future. Research indicates that hope, as a positive psychological trait, is gradually receiving more attention in the management of chronic diseases. Snyder (2002) posits that hope is an internal cognitive assessment mechanism established by individuals to achieve desired goals. According to Snyder’s Hope Theory, hope is a fusion of goal-oriented thinking, pathway thinking, and agency thinking. Goal is the core component of hope theory, and the specific manifestations of individual actions depend on the goals set. Pathway thinking is the cognitive aspect of hope, representing practical skills derived from personal internal psychological planning. Through pathway thinking, individuals can identify paths and strategies to achieve their goals. Agency thinking initiates individual actions, serving as the motivational component of the theory that propels individuals along the designated path toward their goals and sustains their perseverance and resilience in overcoming challenges. Individuals with high levels of hope often have clearer goals, stronger path thinking, and dynamic thinking. They are more inclined to proactively adopt positive coping strategies to deal with problems, thus improving patients’ self-management behavior (Snyder, 2002; Corn et al., 2020). Previous studies have demonstrated a positive correlation between hope and self-management (Veres et al., 2014; Zhang et al., 2023). However, evidence of hope in PCI patients in the Chinese context is lacking. Thus, the relationship among inner strength, hope, and self-management remains unclear.

**FIGURE 1:**
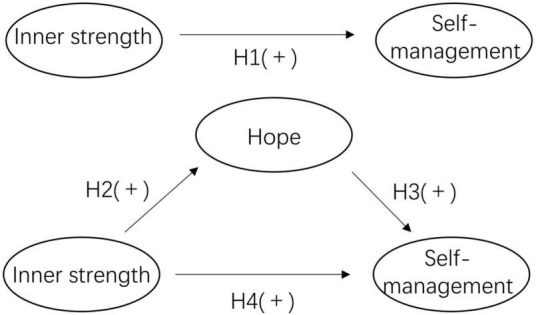
Theoretical model and hypotheses.

Research specifically focused on inner strength among PCI patients, and the associated issues of hope and self-management, are lacking in mainland China. Therefore, one of the objectives of this study was to understand the status of self-management, inner strength, and hope in patients post-PCI. In addition, according to Fredrickson’s Broaden-and-Build Theory of Positive Emotions, positive emotions, such as hope, play a crucial role in establishing enduring internal resources. These resources empower individuals to fully engage their initiative, fostering a range of thoughts and behaviors, particularly those characterized by creativity and innovation (Fredrickson and Branigan, 2005). Aligned with the Broaden-and-Build Theory, the second objective of this study is to elucidate the protective function of positive emotion (hope) as a potential mediator in the relationship between inner strength and self-management behavior among post-PCI patients. The following hypotheses were formulated: Hypothesis 1: Inner strength is positively related to self-management. Hypothesis 2: Inner strength is positively related to hope. Hypothesis 3: Hope is positively related to self-management. Hypothesis 4: Hope plays a mediating role between inner strength and self-management. The proposed model is constructed in Figure 1. The finding will enrich relevant theory and provide a new perspective for self-management in patients after PCI.

## 2 Materials and methods

### 2.1 Study design and procedure

A cross-sectional study was conducted at Nanjing First Hospital from September 2022 to February 2023. Paper questionnaires were distributed by two trained investigators with master’s degrees when the patients were stable. The survey was conducted in the teaching classroom or patient ward of the Cardiology Department at the Nanjing First Hospital. At the start of the survey, the investigators explained the study’s purpose and significance to the participants using unified terminology and informed the patients about the points to consider when completing the questionnaires. With their consent, patients were guided to complete the questionnaire anonymously. Each questionnaire took about 10–15 min to complete and was collected on the spot. Any confusion or mistakes made during the completion of the questionnaire were immediately clarified and corrected. According to the principle that the sample size for multiple linear regression should be at least 5–10 times the number of independent variables, and considering there were about 22 variables in this study, the estimated sample size was set at 220 subjects to account for potential non-respondents (Chow et al., 2018).

### 2.2 Participants

Convenience sampling was employed to recruit participants. Patients who underwent PCI at Nanjing First Hospital were recruited for the study. The inclusion criteria were: (a) at least 18 years old, (b) met the criteria of the Chinese Guidelines for Percutaneous Coronary Interventions (2016) and received PCI for the first time (Han, 2016), (c) able to provide written informed consent, (d) able to complete the questionnaire independently or with the assistance of the investigator. The exclusion criteria were: (a) patients diagnosed with psychiatric diseases, such as schizophrenia, (b) patients with other severe somatic diseases, such as multiple organ failure or malignant tumors, (c) patients unable to take care of themselves in daily life. A total of 220 questionnaires were distributed, and after eliminating the unqualified ones, 216 questionnaires were deemed valid.

### 2.3 Measures

#### 2.3.1 The self-designed general information questionnaire

The collected information included age, gender, marital status, education level, monthly family income, course of coronary heart disease and number of complications.

#### 2.3.2 Inner strength scale

The Inner Strength Scale (ISS), developed by Lundman et al. (2011) based on their metatheoretical analysis, was used to assess the capacity that supports positive movement through life-challenging events. It is a 20-item scale with four dimensions: firmness, creativity, connectivity, and flexibility. The ISS was validated in Chinese among patients living with chronic illness (Hu et al., 2022). Courage was added as a new factor to the original scale, and the revised ISS contained five dimensions with a total of 20 items. Participants rated each item on a 6-point Likert scale (from 1 “completely disagree,” to 6 “completely agree”). The total score of ISS, which ranged from 20 to 120, covered the sum of each item. Higher scores indicated stronger inner strength of patients. ISS has been widely used in the elderly population in China with satisfactory reliability and validity (Yu et al., 2018; Hu et al., 2022). The Cronbach’s alpha coefficient of ISS in the present study was 0.914.

#### 2.3.3 Herth hope index

The Herth Hope Index (HHI), compiled by Herth (1991), was applied to assess the positive attitudes toward uncertain events such as illness. The Chinese version was translated by Chan et al. (2012). HHI consists of 12 items in three dimensions: temporality and future (T), positive readiness and expectancy (P), and interconnectedness (I). Participants rated each item on a 4-point Likert scale (from 1 “completely disagree,” to 4 “completely agree”). The total score of HHI ranged from 12 to 48, and higher scores indicated higher levels of hope. The HHI is suitable for the assessment of hope in healthy populations, cancer, and other chronic diseases (Smith et al., 2019). In addition, the HHI has been demonstrated to be a reliable and valid instrument for evaluating hope in patients with heart disease (Chan et al., 2012). The Cronbach’s alpha coefficient of ISS in the present study was 0.867.

#### 2.3.4 Coronary self-management scale

The Coronary Self-Management Scale (CSMS) was compiled by Ren et al. (2009) and used to evaluate the management of related health behaviors in patients with coronary heart disease in China. The CSMS consists of seven dimensions, including daily life management, adverse habits management, disease knowledge management, symptom management, first aid technology management, treatment compliance management, and emotional cognition management, with a total of 27 items. Participants rated each item on a 5-point Likert scale (from 1 “never,” to 5 “always”). The total score of CSMS ranged from 27 to 135, and higher scores indicated higher levels of self-management. Currently, this scale is widely employed in China for the measurement of self-management behavior in CHD patients with good reliability and validity (Zhang et al., 2021; Zhu et al., 2022). The Cronbach’s alpha coefficient of CSMS in the study was 0.943.

### 2.4 Data analysis

SPSS 26.0 was used for data processing and analysis. Prior to data analysis, the Shapiro–Wilk test was performed to check the normal distribution of the data. Continuous variables (ISS, HHI, CSMS scores) were normally distributed and described by mean ± standard deviation (SD). Categorical variables (age, gender, marital status, education level, monthly family income, course of coronary heart disease, and complications) were represented by frequency and percentage (%). The *t*-test or one-way analysis of variance was used to compare the differences in inner strength, hope, and self-management of CHD patients with different demographic characteristics. Pearson correlation analysis was performed to determine the correlation among inner strength, hope, and self-management in patients with CHD. A two-sided *P* < 0.05 was considered statistically significant. The mediating role of hope in the relationship between inner strength and self-management was tested using the bootstrap method (5,000 runs) by running the PROCESS macro 3.4 (model 4) developed by Hayes (2017) in SPSS. If 95% confidence intervals (CIs) did not include zero, then the effect was regarded as significant.

## 3 Results

### 3.1 Common method bias

Common method bias was tested using Harman’s single-factor test. The results showed that 11 factors with eigenvalues over 1 were obtained by unrotated principal component factor analysis. The variance explained by the first factor was 37.92%, less than the critical value of 40.00%, indicating that there was no serious common method bias in this study.

### 3.2 Comparison of inner strength, hope, and self-management scores in PCI patients with different demographic characteristics

The demographic characteristics of the research variables are shown in Table 1. Of the 216 respondents, 133 (61.6%) were males. The mean age ( ± SD) of the patients was 63.14 ± 10.75 years, ranging from 32 to 86 years. The majority of the participants were married (*n* = 207, 95.8%). About 33.8% of the patients had a middle school education. Half of the patients had a monthly family income ranging from 5,000 to 10,000 yuan (*n* = 108, 50.0%). Approximately 63.8% of the patients had a disease duration of less than 0.5 years. A total of 186 (86.1%) patients had at least one complication. As shown in Table 1, there were significant differences in the scores of hope and inner strength among patients with different genders, ages, education levels, and marital statuses (all *P* < 0.05). There were significant differences in the scores of self-management among patients with different ages, education levels, and disease durations (all *P* < 0.05).

**TABLE 1:** Comparison of ISS, HHI, and CSMS with different demographic characteristics.

Variable	*N* (%)	ISS	HHI	CSMS
**Gender**				
Male	133 (61.6)	82.80 ± 11.32	36.51 ± 5.11	88.24 ± 14.70
Female	83 (38.4)	79.31 ± 12.82	35.01 ± 5.71	84.46 ± 14.86
*t*		2.090	2.004	1.832
*P*		0.038	0.046	0.068
**Age**				
≤45	13 (6.0)	96.62 ± 5.73	43.92 ± 2.40	106.54 ± 11.33
46∼59	70 (32.4)	91.24 ± 3.88	40.20 ± 2.10	96.04 ± 8.19
60∼74	102 (47.2)	79.13 ± 7.58	34.53 ± 3.61	81.21 ± 14.38
≥75	31 (14.4)	60.68 ± 2.10	27.58 ± 1.52	75.97 ± 8.98
*F*		226.753	178.881	42.853
*P*		<0.01	<0.01	<0.01
**Marital status**				
Married	207 (95.8)	82.05 ± 11.76	36.16 ± 5.32	87.11 ± 14.99
Unmarried	2 (1.0)	59.00 ± 0.00	28.50 ± 0.71	79.00 ± 0.00
Divorced/Widowed	7 (3.2)	70.29 ± 9.60	31.43 ± 4.96	79.43 ± 9.74
*F*		7.173	4.694	1.187
*P*		0.001	<0.01	0.307
**Education level**				
Primary school and below	51 (23.6)	76.92 ± 11.32	34.25 ± 4.97	80.45 ± 13.14
Middle school	73 (33.8)	80.29 ± 12.27	35.41 ± 5.50	85.95 ± 16.02
High school or technical secondary school	55 (25.5)	83.07 ± 11.62	36.20 ± 5.14	89.36 ± 13.78
College degree or above	37 (17.1)	87.62 ± 10.22	38.89 ± 5.00	93.35 ± 12.83
*F*		6.740	6.048	6.625
*P*		<0.01	0.001	<0.01
**Monthly family income (RMB)**				
<5,000	58 (26.9)	80.07 ± 11.07	35.38 ± 4.59	83.98 ± 15.64
5,000∼10,000	108 (50.0)	80.72 ± 12.73	35.61 ± 5.84	87.28 ± 15.02
>10,000	50 (23.1)	84.66 ± 11.05	37.28 ± 5.09	88.98 ± 13.22
*F*		2.400	2.085	1.650
*P*		0.093	0.127	0.194
**Disease course (year)**				
<0.5	138 (63.8)	80.88 ± 12.05	35.58 ± 5.47	85.13 ± 13.85
0.5∼1.0	47 (21.8)	80.17 ± 12.09	35.60 ± 5.12	87.32 ± 15.67
>1.0	31 (14.4)	86.00 ± 11.00	38.03 ± 5.06	93.35 ± 16.38
*F*		2.692	2.791	4.037
*P*		0.070	0.064	0.019
**Comorbidities (individual)**				
0	30 (13.9)	81.00 ± 13.59	36.43 ± 6.28	84.60 ± 15.56
1∼2	183 (84.7)	81.53 ± 11.85	35.88 ± 5.27	87.13 ± 14.83
≥3	3 (1.4%)	81.67 ± 6.81	34.33 ± 3.22	87.67 ± 7.57
*F*		0.025	0.269	0.378
*P*		0.975	0.764	0.686

ISS, Inner Strength Scale; HHI, Herth Hope Index; CSMS, Coronary Self-Management Scale.

### 3.3 Correlations between inner strength, hope, and self-management among patients after PCI

The mean scores of ISS, HHI, and CSMS were 81.46 ± 12.00, 35.94 ± 5.38, and 86.79 ± 14.84, respectively. As shown in Table 2, Pearson correlation analysis revealed that the total score of ISS was significantly and positively correlated with the total scores of HHI and CSMS (*r* = 0.867 and 0.630, respectively; all *P* < 0.05). Additionally, the total score of HHI was significantly and positively correlated with the total score of CSMS (*r* = 0.671, *P* < 0.05).

**TABLE 2:** Correlation between ISS, HHI, and CSMS in patients post-PCI.

Variable	ISS	HHI	CSMS
ISS	1		
HHI	0.867^a^	1	
CSMS	0.630^a^	0.671^b^	1

ISS, Inner Strength Scale; HHI, Herth Hope Index; CSMS, Coronary Self-Management Scale.

*^a^*P < 0.05;

*^b^*P < 0.01.

### 3.4 Analysis of the mediating effects of hope between inner strength and self-management

**FIGURE 2:**
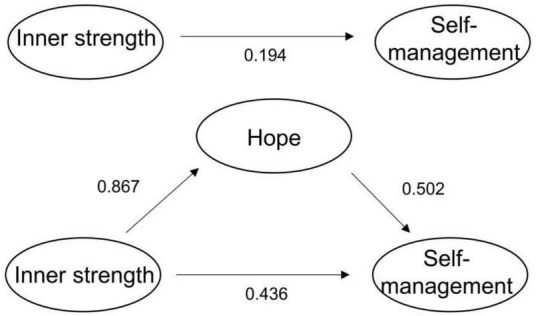
A mediation model depicting the impact of hope in the relationship between inner strength and self-management among patients after PCI.

As shown in Table 3, the first step was to examine the effect of inner strength on self-management. The result showed that inner strength had a significant effect on self-management (β = 0.630, *P* < 0.01). The second step was to examine the effect of inner strength on hope. The result showed that inner strength had a significant effect on hope (β = 0.867, *P* < 0.01). The third step was to include inner strength and hope as independent variables in the regression equation to examine their impact on self-management. The result showed that the influence of inner strength on self-management was not significant after introducing hope as a mediating variable (β = 0.194, *P* = 0.057), suggesting that hope had a complete mediating effect between inner strength and self-management. To further determine whether the mediating effect coefficient was significant, the Bootstrap method was used to verify the confidence interval of the mediating effect. Five thousand samples were randomly sampled from the original data (*n* = 216), and the average of the path coefficient was calculated. As shown in the Table 4, the 95% confidence interval of the direct effect of inner strength on self-management contains 0 (−0.006∼0.394), while the 95% confidence interval of the mediating effect of hope does not contain 0 (0.285∼0.601), indicating that the mediation effect of hope is significant and completely mediating. The mediating effect (0.436) accounts for 69.21% of the total effect (0.630). The mediation effect model is shown in Figure 2.

**TABLE 3:** Analysis of mediating effect of hope between inner strength and self-management.

Procedure	Dependent variable	Independent variable	*R* ^2^	β value (SE)	*t* value	*P*-value
Step 1	CSMS	ISS	0.396	0.630 (0.053)	11.857	<0.01
Step 2	HHI	ISS	0.752	0.867 (0.034)	25.485	<0.01
Step3	CSMS	ISS	0.459	0.194 (0.101)	1.916	0.057
		HHI		0.502 (0.101)	4.963	<0.01

ISS, Inner Strength Scale; HHI, Herth Hope Index; CSMS, Coronary Self-Management Scale.

**TABLE 4:** Outcomes of the mediation models.

Path	Effect value	SE	95% CI	Relative effect value
Direct effect (c’): ISS → CSMS	0.194	0.101	−0.006∼0.394	30.79
Mediating effect (a × b): ISS → HHI → CSMS	0.436	0.080	0.285∼0.601	69.21
Total effect (c): ISS → HHI	0.630	0.053	0.525∼0.734	100.00

ISS, Inner Strength Scale; HHI, Herth Hope Index; CSMS, Coronary Self-Management Scale; 95% CI, 95% confidence interval.

## 4 Discussion

This study investigated the status of self-management, hope, and inner strength in patients after PCI and, to our knowledge, for the first time, tested the mediating role of hope between inner strength and self-management. Several analyses were carried out for the research question, including descriptive statistics, correlation, and mediation analysis.

Our findings revealed that self-management of patients after PCI was at a moderate level (86.79 ± 14.84), which was similar to the results obtained by Ma et al. (2018) in the study of self-management of patients after PCI in China. General life management had the highest item average score (3.38 ± 0.68), while emotional cognition management had the lowest item average score (2.98 ± 0.77), followed by treatment compliance management (3.02 ± 0.70) and bad habit management (3.17 ± 0.66). These results indicate that compared with understanding a healthy diet and appropriate activities in daily life, patients after PCI have more difficulties in long-term self-management behavior, changing bad habits such as smoking, and emotional regulation after an illness. This suggests that medical workers and family members should strengthen the supervision of the health behavior of such patients, promote the improvement of compliance management, and also pay attention to psychological counseling (Zhu et al., 2022). In addition, the difference analysis of self-management scores of patients with different socio-demographic characteristics showed that the higher the education level and the longer the course of the disease, the higher the level of self-management scores. This was consistent with previous studies (Lai et al., 2021). However, this study showed a negative association between age and self-management, different from the umbrella review results of Alexandre, which may be related to the relatively high literacy of younger patients in this study (Alexandre et al., 2021). On the one hand, compared with older patients, younger patients have better physical function and find it easier to complete the management of symptoms and general life. On the other hand, younger, more educated patients with a longer disease course may have a better ability to acquire, understand, and apply health-related information, more experience in coping with the impact of the disease, and therefore better self-management ability (Guo and Harris, 2016; Alexandre et al., 2021; Lai et al., 2021).

To our knowledge, there has been no quantitative study on inner strength in patients after PCI. In this study, the ISS of patients was (81.46 ± 12.00), which was at the medium level, similar to the chronic population (Hu et al., 2022). However, it was lower than Viglund’s findings in the healthy elderly group (Viglund et al., 2013). Compared with healthy people, patients with CHD bear more burden of symptoms and economic pressure brought by the disease. Especially for those who undergo PCI for the first time, more attention is paid to treatment effect and prognosis in the short term, so it is easy to overlook the existence and application of individual internal resources. In this study, the HHI of patients was (35.94 ± 5.38), similar to the results of Zhang, and higher than that of the tumor population (Li et al., 2021; Zhang et al., 2021; Wnuk, 2022). As a Chinese saying goes, “Turn pale at the mention of cancer,” influenced by traditional concepts, patients have less fear of CHD, which is a common chronic disease in their mind, than cancer. In addition, most patients in this study had a disease course within 1 year after PCI (185 cases, 85.6%), so they have greater expectations for prognosis and control of disease progression. The present study found that patients’ inner strength and hope scores after PCI varied by gender, age, education level and marital status. Compared with males, females are more psychologically vulnerable and more likely to have negative emotions, so their internal resources are relatively weak (Shimamoto and Rappeneau, 2017). Moreover, high educational level and married status have a maintenance and promoting effect on inner strength and hope, which may be related to the fact that such patients have more scientific channels to acquire disease knowledge, better self-regulation ability, more social support and perception, and thus have more belief and hope to overcome the disease (Viglund et al., 2013).

Correlation analysis showed that there was a positive correlation between the inner strength score and self-management score of patients after PCI (*r* = 0.630, *P* < 0.01). This result adds quantitative evidence to the view that the improvement of the self-management ability of chronic patients is the result of inner strength in the theoretical model of inner strength proposed by Dingley and Roux (2014). According to Dingley’s theoretical model, patients with higher inner strength can face up to challenging life events and learn from the experience in the process of illness. By establishing connections with themselves, family and friends, the patients continuously optimize the use of internal and external resources, and adopt familiar behaviors (such as changing their diet, reasonable exercise, adherence to medication, etc.) to make positive self-adjustment to reach a new normal. The results of this study showed that the inner strength of patients after PCI was positively correlated with the level of hope, which was similar to the results of Borges et al. (2021). Patients with higher levels of inner strength are more inclined to view life after illness from a positive perspective, thus more likely to adjust their mentality to accept the disease and have more confidence and hope for physical and mental recovery (Zhang, 2021). The results of this study show that patients’ hope level is positively associated with self-management, indicating that patients with high hope levels are more likely to adhere to treatment and effectively self-manage, which is consistent with previous findings (Snyder, 2002). As a positive internal dynamic force, hope is an important strategy for patients to cope with the disease. Existing studies show that a good level of hope can mobilize people’s positive emotions and self-efficacy (Davidson et al., 2020). Patients with a higher level of hope are better able to resist the harm brought by negative emotions and have expectations and persistence for the treatment and rehabilitation of the disease. Therefore, they have a strong belief in the successful implementation and completion of a certain behavioral goal of self-management, which makes patients more willing to actively learn and cooperate with others to take positive actions to deal with the disease and improve their physical condition. Based on the above results, healthcare providers should value the positive significance of intrinsic strength and hope for patient behavior change. Nursing staff can encourage patients to face up to the disease through health education or psychological intervention, correct their misconceptions such as “illness = disability” and “PCI = cure,” and help patients realize the role of individual power in delaying the disease process, so as to improve the plasticity and enthusiasm of patients’ self-management.

In this study, the analysis of mediation effects showed that the level of hope is a mediator variable of inner strength and self-management and acts as a complete mediator. This indicates that inner strength after PCI can influence and predict patients’ self-management ability through the level of hope. Patients with higher inner strength have more core resources to resist disease and show stronger psychological adaptation and spiritual growth in the process of coping with the disease. Patients with inner strength do not deny the fact of illness, but regard the disease as normal, and have hope for the treatment and recovery of the disease in the process of actively accepting the disease. Driven by the positive spiritual force of hope, the patient’s health responsibility is stimulated, and they are more inclined to take the initiative to deal with negative emotions, make use of social resources, and exercise self-control to achieve good self-management. Therefore, while providing objective support such as health knowledge and medical care for patients after PCI, medical staff should pay attention to the positive effect of patients’ psychological capital on behavior. According to Snyder’s hope theory, a nursing plan can be formulated from three aspects: goal, path thinking, and dynamic thinking (Ge et al., 2021). For example, when developing self-care strategies for patients, the whole process should be phased, and the patients can jointly agree on phased goals, starting from simple short-term goals, so that patients can have a positive experience of “I can” in the completion process. They can adjust their cognition of disease events, and treat disease as an opportunity to pay attention to their own health and make positive changes, encourage patients to pay attention to their own value in health promotion, and help patients to explore and use their own advantages to actively participate in the self-management of disease.

## 5 Limitations of the study

While the results of this study strongly supported the hypothesis of the mediating role of hope between inner strength and self-management, several limitations of the study must be acknowledged. First, this study adopts a cross-sectional design, so the causal relationship between variables should be considered carefully. Future research could conduct longitudinal studies to further verify the results of this model. Second, all the samples in this study were from a third-class A hospital in Nanjing, Jiangsu Province, which has the leading percutaneous coronary intervention technology in China. Patients who sought treatment here may have relatively high levels of hope and self-management awareness, leading to a certain degree of sampling bias. Therefore, the results of this study may not be extended to other coronary heart disease populations. Future research needs to be conducted on a larger scale and in multiple locations. Third, the disease course of most patients included in this study is within half a year, and the survival time of patients with the disease is not long. With the passage of time, patients’ views on disease and disease management may change. Therefore, it is necessary to conduct longitudinal studies to explore the dynamic changes in patients’ psychological experience and self-management. Moreover, this study only analyzed the relationship between inner strength, hope, and self-management through a simple mediation model. The exploration of mechanisms is far from sufficient, and in the future, more scientific approaches such as mediation chains, moderation effects, etc., should be employed for a more comprehensive and in-depth investigation into the mechanisms through which inner strength influences self-management.

## 6 Conclusion

This study demonstrated that the self-management, inner strength, and hope of patients after PCI were all at a moderate level, and there was still room for further improvement. The concepts were pairwise correlated, and hope played a complete mediating role between inner strength and self-management. First of all, self-management health education for PCI patients still needs to be strengthened, especially for the elderly, the patients with low education and a short course of disease. In addition, there are currently only a few interventional studies on patients’ inner strength. In the future, while exploring intervention strategies related to inner strength, medical staff may take improving patients’ hope level as the perspective and intervention target, so as to help patients rekindle their hope for life, accumulate internal resources, and actively participate in self-management.

## Data availability statement

The original contributions presented in this study are included in this article/supplementary material, further inquiries can be directed to the corresponding author.

## Ethics statement

The studies involving humans were approved by the Ethics Committee of Nanjing First Hospital, Nanjing Medical University. The studies were conducted in accordance with the local legislation and institutional requirements. Written informed consent for participation was not required from the participants or the participants’ legal guardians/next of kin in accordance with the national legislation and institutional requirements.

## Author contributions

SHS: Conceptualization, Data curation, Formal analysis, Investigation, Methodology, Project administration, Writing—original draft, Writing—review and editing. XZ: Supervision, Writing—review and editing, Validation. ZX: Data curation, Investigation, Writing—review and editing. SS: Formal analysis, Validation, Writing—review and editing. TH: Conceptualization, Supervision, Validation, Writing—review and editing.

## References

[B1] Aggarwal M. Ornish D. Josephson R. Brown T. M. Ostfeld R. J. Gordon N. (2021). Closing gaps in lifestyle adherence for secondary prevention of coronary heart disease. *Am. J. Cardiol.* 145 1–11. 10.1016/j.amjcard.2021.01.005 33454343

[B2] Alexandre K. Campbell J. Bugnon M. Henryet C. Schaub C. Serexal M. (2021). Factors influencing diabetes self-management in adults: an umbrella review of systematic reviews. *JBI Evid. Synth.* 19 1003–1118. 10.11124/JBIES-20-00020 33741836

[B3] Boman E. Häggblom A. Lundman B. Nygren B. Fischer R. S. (2015). Inner strength as identified in narratives of elderly women: a focus group interview study. *ANS Adv. Nurs. Sci.* 38 7–19. 10.1097/ANS.0000000000000057 25635502

[B4] Borges C. C. Santos P. R. Alves P. M. Borgeset R. C. Lucchettial G. Barbosa M. A. (2021). Association between spirituality/religiousness and quality of life among healthy adults: a systematic review. *Health Qual. Life Outcomes* 19:246. 10.1186/s12955-021-01878-7 34674713 PMC8529786

[B5] Chan K. S. Li H. C. Chan S. W. Lopez V. (2012). Herth hope index: psychometric testing of the Chinese version. *J. Adv. Nurs.* 68 2079–2085. 22111952 10.1111/j.1365-2648.2011.05887.x

[B6] Cheng L. Kotronoulas G. (2020). How effective are self-management interventions in promoting health-related quality of life in people after primary treatment for breast cancer? a critical evidence synthesis. *Eur. J. Oncol. Nurs.* 47:01776. 10.1016/j.ejon.2020.101776 32570063

[B7] Chow S. C. Shao J. Wang H. Lokhnygina Y. (2018). *Sample Size Calculations in Clinical Research.* Boca Raton: Chapman and Hall.

[B8] Collins S. P. Liu D. Jenkins C. A. Storrow A. B. Levy P. D. Pang P. S. (2021). Effect of a self-care intervention on 90-day outcomes in patients with acute heart failure discharged from the emergency department: a randomized clinical trial. *JAMA Cardiol.* 6 200–208. 10.1001/jamacardio.2020.5763 33206126 PMC7675219

[B9] Corn B. W. Feldman D. B. Wexler I. (2020). The science of hope. *Lancet Oncol.* 21 e452–e459. 32888474 10.1016/S1470-2045(20)30210-2

[B10] Davidson A. B. McLeigh J. D. Katz C. (2020). Perceived collective efficacy and parenting competence: the roles of quality of life and hope. *Fam. Process.* 59 273–287. 10.1111/famp.12405 30403404

[B11] Dingley C. Bush H. Roux G. (2001). Inner strength in women recovering from coronary artery disease: a grounded theory. *J. Theory Constr. Test.* 5 45–52.

[B12] Dingley C. Roux G. (2014). The role of inner strength in quality of life and self-management in women survivors of cancer. *Res. Nurs. Health* 37 32–41. 10.1002/nur.21579 24357538 PMC4126601

[B13] Feldman D. B. Sills J. R. (2013). Hope and cardiovascular health-promoting behaviour: education alone is not enough. *Psychol. Health* 28 727–745. 10.1080/08870446.2012.754025 23289597

[B14] Fredrickson B. L. Branigan C. (2005). Positive emotions broaden the scope of attention and thought-action repertoires. *Cogn. Emot.* 19 313–332. 21852891 10.1080/02699930441000238 PMC3156609

[B15] Freites A. Hernando L. Salinas P. Cánovaset E. Rosa A. Alonsoal J. (2022). Incidence and prognosis of late readmission after percutaneous coronary intervention. *Cardiol. J.* Epub ahead of print. 10.5603/CJ.a2022.0117 36510791 PMC10635725

[B16] Gao R. L. (2019). Advances and perspectives in coronary intervention in China. *Chin. J. Cardiol.* 47 675–679. 10.3760/cma.j.issn.0253-3758.2019.09.002 31550832

[B17] Ge C. Zhang H. Zhu G. Cao A. P. Zhang J. P. (2021). Intervention study of Snyder’s hope theory on the stigma of stroke in young and middle-aged patients: a randomised trial. *Ann. Palliat. Med.* 10 5721–5728. 10.21037/apm-21-441 33977743

[B18] Gottlieb L. N. (2014). Strengths-based nursing. *Am. J. Nurs.* 114 24–32. 10.1097/01.NAJ.0000453039.70629.e2 25036663

[B19] Guo P. Harris R. (2016). The effectiveness and experience of self-management following acute coronary syndrome: a review of the literature. *Int. J. Nurs. Stud.* 61 29–51. 10.1016/j.ijnurstu.2016.05.008 27267181

[B20] Han Y. L. (2016). Interpretation of the Chinese guidelines for percutaneous coronary intervention treatment (2016). *Chin. Circulation J.* 31 5–8.

[B21] Hayes A. F. (2017). *Introduction to Mediation, Moderation, and Conditional Process Analysis: a Regression-Based Approach.* New York, NY: Guilford publications.

[B22] Herth K. (1991). Development and refinement of an instrument to measure hope. *Sch. Inq. Nurs. Pract.* 5 39–51. 2063043

[B23] Hirao Y. Seki T. Watanabe N. Matoba S. (2023). Health-related quality of life after percutaneous coronary intervention for stable ischemic heart disease: a systematic review and meta-analysis. *Can. J. Cardiol.* 39 1539–1548. 37422259 10.1016/j.cjca.2023.06.429

[B24] Hoole S. P. Bambrough P. (2020). Recent advances in percutaneous coronary intervention. *Heart* 106 1380–1386. 32522821 10.1136/heartjnl-2019-315707

[B25] Hu H. J. Guo X. Q. Tang Q. Q. Cheng J. Li H. Y. Li T. T. (2022). Mediating effect of cognition of aging between perceived social support and inner strength of the elderly in nursing homes. *J. Nurs.* 29 57–61.

[B26] Huber K. Ulmer H. Lang I. Mühlberger V. (2020). Coronary interventions in Austria, Germany, and Switzerland. *Eur. Heart J.* 41 2599–2600. 32385514 10.1093/eurheartj/ehaa291

[B27] Knuuti J. Wijns W. Saraste A. Capodannoet D. Barbatoal E. Funck-Brentano C. (2020). 2019 ESC Guidelines for the diagnosis and management of chronic coronary syndromes. *Eur. Heart J.* 41 407–477. 31504439 10.1093/eurheartj/ehz425

[B28] Kumsa N. B. Kelly T. L. Roughead E. E. Tavella R. Gillam M. H. (2023). Temporal trends in percutaneous coronary intervention in Australia: a retrospective analysis from 2000-2021. *Hellenic J. Cardiol.* Online ahead of print. 10.1016/j.hjc.2023.10.002 37863429

[B29] Lai P. C. Wu S. V. Alizargar J. Pranata S. Tsai J. M. Hsiehet N. C. (2021). Factors influencing self-efficacy and self-management among patients with Pre-End-Stage Renal Disease (Pre-ESRD). *Healthcare* 9:266. 10.3390/healthcare9030266 33801477 PMC8000963

[B30] Li Y. Ni N. Zhou Z. Dong J. Y. Fu Y. Li J. X. (2021). Hope and symptom burden of women with breast cancer undergoing chemotherapy: a cross-sectional study. *J. Clin. Nurs.* 30 2293–2300. 10.1111/jocn.15759 33756013

[B31] Liu J. T. Su H. Qin X. J. Lan Y. X. Zhang J. Z. (2023). Guidelines on cardiac rehabilitation in patients with coronary heart disease:a systematic review. *Chin. General Med.* 26 2323–2331.

[B32] Liu S. Li Y. Zeng X. Wang H. Yin P. Wang L. (2019). Burden of cardiovascular diseases in China, 1990-2016: findings from the 2016 global burden of disease study. *JAMA Cardiol.* 4 342–352. 10.1001/jamacardio.2019.0295 30865215 PMC6484795

[B33] Lloyd-Jones D. Adams R. J. Brown T. M. Carnethon M. Dai S. De Simone G. (2010). American heart association statistics committee and stroke statistics subcommittee. heart disease and stroke statistics–2010 update: a report from the American heart association. *Circulation* 121 e46–e215. 20019324 10.1161/CIRCULATIONAHA.109.192667

[B34] Lundman B. Viglund K. Aléx L. Jonsénet E. Norbergal A. Fischer R. S. (2011). Development and psychometric properties of the inner strength scale. *Int. J. Nurs. Stud.* 48 1266–1274. 21474137 10.1016/j.ijnurstu.2011.03.006

[B35] Ma H. Yang F. G. Lu X. H. Xia T. T. Yin C. L. Li Y. Y. (2018). Influence factors of self-management behavior of patients with percutaneous coronary intervention. *J. Nurs.* 25 1–4.

[B36] Mendes B. Roux G. Ridosh M. (2010). Phenomenon of inner strength in women post-myocardial infarction. *Crit Care Nurs Q.* 33 248–258. 10.1097/CNQ.0b013e3181e6d809 20551739

[B37] Müller R. Segerer W. Ronca E. Gemperli A. Stirnimann D. Scheel-Sailer A. (2022). Inducing positive emotions to reduce chronic pain: a randomized controlled trial of positive psychology exercises. *Disabil. Rehabil.* 44 2691–2704. 10.1080/09638288.2020.1850888 33264568

[B38] Orem D. E. (1981). Nursing: concepts of practice. *Aorn J.* 34 776–776.

[B39] Pan F. (2023). The interventional treatment of heart disease in China is showing a positive development trend. *China Med. Herald.* 20 1–3.

[B40] Peterson J. C. Link A. R. Jobe J. B. Winston G. J. Marina Klimasiewfski E. Allegrante J. P. (2014). Developing self-management education in coronary artery disease. *Heart Lung*. 43, 133–139. 10.1016/j.hrtlng.2013.11.006 24373484 PMC3947696

[B41] Ren H. Y. Tang P. Zhao Q. H. (2009). Development and evaluation of coronary artery disease self-management scale. *J. Army Med. Univ.* 31 1087–1090.

[B42] Riegel B. Jaarsma T. Strömberg A. (2012). A middle-range theory of self-care of chronic illness. *ANS Adv. Nurs. Sci.* 35 194–204. 22739426 10.1097/ANS.0b013e318261b1ba

[B43] Roth G. A. Mensah G. A. Johnson C. O. Addolorato G. Ammirati E. Baddour L. M. (2020). Global burden of cardiovascular diseases and risk factors, 1990-2019: update from the GBD 2019 study. *J. Am. Coll. Cardiol.* 76 2982–3021. 33309175 10.1016/j.jacc.2020.11.010 PMC7755038

[B44] Roux G. Dingley C. E. Bush H. A. (2002). Inner strength in women: metasynthesis of qualitative findings in theory development. *J. Theory Constr. Test* 6 86–93.

[B45] Shimamoto A. Rappeneau V. (2017). Sex-dependent mental illnesses and mitochondria. *Schizophr. Res.* 187 38–46. 28279571 10.1016/j.schres.2017.02.025 PMC5581986

[B46] Smith C. S. Dingley C. Roux G. (2019). Inner strength-state of the science. *Can. J. Nurs. Res.* 51 38–48. 10.1177/0844562118790714 30053785

[B47] Snyder C. (2002). Hope theory: rainbows in the mind. *Psychol. Inq.* 13 249–275.

[B48] Sua Y. S. Jiang Y. Thompson D. R. Wang W. R. (2020). Effectiveness of mobile phone-based self-management interventions for medication adherence and change in blood pressure in patients with coronary heart disease: a systematic review and meta-analysis. *Eur. J. Cardiovasc. Nurs.* 19 192–200. 10.1177/1474515119895678 31856596

[B49] Tsao C. W. Aday A. W. Almarzooq Z. I. Anderson C. A. M. Arora P. Avery C. L. (2023). American heart association council on epidemiology and prevention statistics committee and stroke statistics subcommittee. heart disease and stroke statistics-2023 update: a report from the American heart association. *Circulation* 147 e93–e621. 10.1161/CIR.0000000000001123 36695182 PMC12135016

[B50] Veres A. Bain L. Tin D. Thorne C. Ginsburg L. R. (2014). The neglected importance of hope in self-management programs - a call for action. *Chronic Illn.* 10 77–80. 10.1177/1742395313496827 24821605

[B51] Viglund K. Jonsén E. Lundman B. Strandberg G. Nygren B. (2013). Inner strength in relation to age, gender and culture among old people–a cross-sectional population study in two Nordic countries. *Aging Ment. Health* 17 1016–1022. 10.1080/13607863.2013.805401 23750849

[B52] Viglund K. Jonsén E. Strandberg G. Lundman B. Nygren B. (2014). Inner strength as a mediator of the relationship between disease and self-rated health among old people. *J. Adv. Nurs.* 70 144–152. 10.1111/jan.12179 23718213

[B53] Wnuk M. (2022). Beneficial effects of spiritual experiences and existential aspects of life satisfaction of breast and lung cancer patients in poland: a pilot study. *J. Relig. Health* 61 4320–4336. 10.1007/s10943-022-01601-w 35748968 PMC9569296

[B54] World Health Organization [WHO] (2002). *Innovative Care for Chronic Conditions: Building Blocks for Action, Noncommunicable and Mental Health.* Geneva: World Health Organization.

[B55] Writing Committee of the Report on Cardiovascular Health and Diseases in China (2022). Report on cardiovascular health and diseases in China 2021: an updated summary. *Biomed. Environ. Sci.* 35 573–603.35945174

[B56] Yu Y. Z. Li D. Liu H. Q. Mai J. Y. Dai L. Wang D. H. (2018). Study on the correlation between death attitudes and inner strength in elderly patients with chronic diseases in rural areas. *J. Nurs. Adm.* 18 193–197.

[B57] Zhang D. Zhang N. Chang H. Shi Y. Tao Z. Zhang X. (2023). Mediating role of hope between social support and self-management among chinese liver transplant recipients: a multi-center cross-sectional study. *Clin. Nurs. Res.* 32 776–784. 10.1177/10547738221078897 35195036

[B58] Zhang X. Chen H. Liu Y. Yang B. (2021). Influence of chronic illness resources on self-management and the mediating effect of patient activation among patients with coronary heart disease. *Nurs. Open* 8 3181–3189. 34498405 10.1002/nop2.1031 PMC8510723

[B59] Zhang X. H. (2021). Study on the correlation between social support, and level of hope, negative emotion, and compliance behavior in patients with coronary heart disease after PCI. *J. Bengbu Med. Coll.* 46 1141–1145.

[B60] Zhao D. Liu J. Wang M. Zhang X. G. Zhou M. G. (2019). Epidemiology of cardiovascular disease in China: current features and implications. *Nat. Rev. Cardiol.* 16 203–212. 10.1038/s41569-018-0119-4 30467329

[B61] Zhu H. X. Chen G. H. Xue X. H. Zheng S. F. (2022). Self-management in patients with coronary heart disease after stent implantation at the long-term stage: a cross-sectional study. *Ann. Palliat. Med.* 11 2265–2274. 10.21037/apm-21-2465 35445603

